# Survival of adult neurons lacking cholesterol synthesis *in vivo*

**DOI:** 10.1186/1471-2202-8-1

**Published:** 2007-01-02

**Authors:** Ursula Fünfschilling, Gesine Saher, Le Xiao, Wiebke Möbius, Klaus-Armin Nave

**Affiliations:** 1 Department of Neurogenetics, Max-Planck-Institute of Experimental Medicine, Hermann-Rein Strasse 3, D-37075 Göttingen, Germany

## Abstract

**Background:**

Cholesterol, an essential component of all mammalian plasma membranes, is highly enriched in the brain. Both during development and in the adult, brain cholesterol is derived from local cholesterol synthesis and not taken up from the circulation. However, the contribution of neurons and glial cells to total brain cholesterol metabolism is unknown.

**Results:**

Using conditional gene inactivation in the mouse, we disrupted the squalene synthase gene (*fdft1*), which is critical for cholesterol synthesis, in cerebellar granule cells and some precerebellar nuclei. Mutant mice showed no histological signs of neuronal degeneration, displayed ultrastructurally normal synapses, and exhibited normal motor coordination. This revealed that these adult neurons do not require cell-autonomous cholesterol synthesis for survival or function.

**Conclusion:**

We conclude that at least some adult neurons no longer require endogenous cholesterol synthesis and can fully meet their cholesterol needs by uptake from their surrounding. Glia are a likely source of cholesterol in the central nervous system.

## Background

Cholesterol is an essential, integral membrane component of mammalian plasma membranes. Genetic or pharmacological interference with cholesterol synthesis during development has dramatic effects on mice, ranging from embryonic lethality to severe brain abnormalities including holoprosencephaly [1-3]. Defects of cholesterol homeostasis in the adult brain are linked to neurodegenerative diseases like Niemann-Pick type C disease or Alzheimer's disease [4-6] or even to general synaptic degeneration [7].

Several studies investigated total cholesterol production in and excretion by the brain [8,9], but very little is known about the contribution of individual cell types. Oligodendrocytes, which require large amounts of cholesterol for the production of myelin, produce most of the cholesterol cell-autonomously [10,11]. Astrocytes are assumed to be net cholesterol producers, as they express ApoE *in vivo *and can secrete cholesterol in ApoE-containing particles *in vitro *[reviewed in [12]]. Cultured neurons from different sources can grow neurites in the absence of added cholesterol, but in cultured retinal ganglion cells, cholesterol was shown to be rate-limiting for synaptogenesis [13]. Such cell culture experiments led to the hypothesis that some developing neurons produce their own cholesterol, but switch to astroglial cholesterol supply at later stages. However, *in vivo *evidence for this hypothesis is missing [14]. We therefore set forth to study the role of neuronal cholesterol synthesis in the adult brain.

We recently generated a mouse line with a conditional null allele of the squalene synthase gene (fdft1^flox/flox^) [11]. Squalene synthase catalyzes the first committed step in sterol biosynthesis, and the complete knockout displays an early embryonic lethal phenotype [2]. In the conditional mouse line, an essential exon of the squalene synthase gene is flanked by loxP site. When crossed to a mouse line that expresses cre recombinase in a cell-type specific manner, this leads to the cell-type specific deletion of squalene synthase and thus cholesterol production. When squalene synthase is eliminated in myelinating glia cells, mutant animals show severe hypomyelination and one third of the animals die before weaning [11]. When squalene synthase is deleted embryonically in nestin-positive neuronal and glial precursors [15], no viable pups are born, highlighting the importance of brain cholesterol production during development (data not shown). To target adult neurons, we chose the mouse line Tg(mα6-cre)B1LFR, which expresses cre recombinase specifically in postmitotic, postmigratory cerebellar granule cells and a few brainstem nuclei [16]. In contrast to the above-mentioned examples, these mutant animals developed normally and were indistinguishable from control littermates up to at least the age of six months. This demonstrates that some adult neurons do not require continuous cell-autonomous cholesterol synthesis and suggests that they survive utilizing horizontal cholesterol flux *in vivo*.

## Results

**Figure 1:**
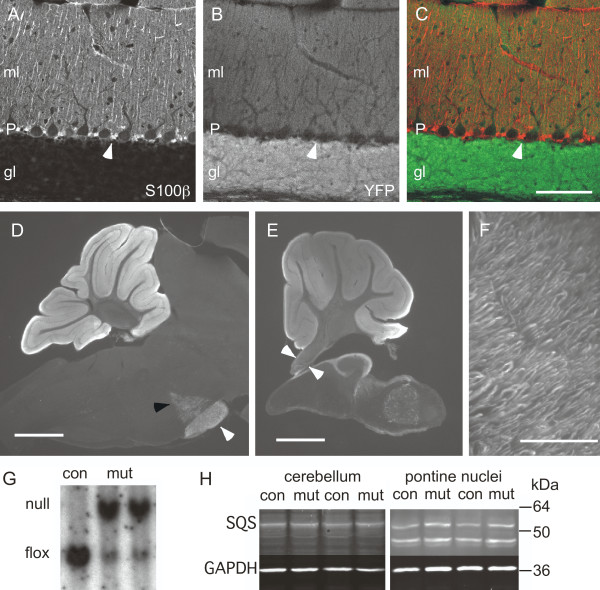
**Conditional deletion of the *fdft1 *gene in vivo**. (A-F) Expression of cre-recombinase in transgenic mice (Tg(mα6-cre)B1LFR) is illustrated by expression of yellow fluorescent protein (YFP) from the R26R-EYFP reporter allele. Vibratome sections of adult cerebellum were double-labeled for the Bergmann glia marker S100β (in A) and the recombination marker YFP (in B). Purkinje neurons (P) and interneurons in the molecular layer (ml) are identified as unstained "holes" (in A-C). YFP is exclusively expressed in granule cells of the granule cell layer (gl) and in their axons that form a weakly stained "lawn" of parallel fibers in the molecular layer. The merge in (C) highlights the lack of YFP expression in Bergmann glia (in red, arrow head). A single confocal plane is depicted, scale bar = 100 μm. (D-E) Overview of targeted cells in a triple transgenic mutant mouse (fdft1^flox/flox^, Tg(mα6-cre)B1LFR, YFP-reporter). Staining vibratome sections from an adult animal for YFP reveals that cerebellar granule cells, pontine nuclei (white arrow head in E) and reticulotegmental nucleus of the pons (black arrow head in E) survive into adulthood in the absence of squalene synthase. The YFP-positive axons from the targeted pontine neurons enter the cerebellum via the middle cerebellar peduncle (arrowheads in E) and blowup (in F). scale bar = 1 mm (in D-E) and 50 μm (in F). (G) 80 % of all cells in the cerebellum are recombined. Southern blot on total cerebellar DNA from a control (con) and mutant (mut) animals, age two months, was hybridized to a probe that recognizes distinct fragment sizes for the flox allele (flox) and the recombined null allele (null). (F) The level of squalene synthase protein is not significantly reduced *in vivo *at postnatal day 16. A western blot of total cerebellar lysate (cerebellum) or the pontine nuclei from two controls (con) and two mutants (mut) was probed for squalene synthase (SQS) and glyceraldeyde-3-phosphate dehydrogenase (GAPDH) as a loading control. Apparent molecular weights are indicated on the right. Squalene synthase was consistently detected as a doublet in the pontine nuclei, but not in cerebellum or cortex (data not shown).

To obtain mutant mice that lack cell-autonomous cholesterol synthesis in defined populations of adult neurons, we crossed the conditional squalene synthase mice fdft1^flox/flox ^[11] with Tg(mα6-cre)B1LFR mice [16] that express cre recombinase postnatally in some precerebellar nuclei and in postmigratory cerebellar granule cells, but not in other cerebellar neurons. To confirm that Bergmann glia, the major cerebellar "astrocytes", are not affected, we reanalyzed Tg(mα6-cre)B1LFR mice crossed to the reporter mice R26R-EYFP [17] that express yellow fluorescent protein (YFP) upon cre-mediated recombination. On vibratome sections of adult cerebellum, double labeling for the Bergmann glia marker S100β and the recombination marker YFP did not reveal any cre expression in glial cells (Figure 1A–C).

Mutant and control animals were obtained at the expected Mendelian ratios and were viable and fertile. Preliminary examination of mutant animals that harbored additionally the R26R-EYFP reporter allele revealed that cerebellar granule cells, the pontine nuclei, and the reticulotegmental nucleus of the pons were present and expressed GFP (Figure 1D–E). The majority of the fibers in the middle cerebellar peduncle, the tract carrying the axons form the pontine nuclei and the reticulotegmental nucleus of the pons to the cerebellar hemispheres, were YFP-positive, indicating that a large fraction of the pontine precerebellar neurons were targeted and had proper projections into the cerebellum (Figure 1E–F). This suggested that the targeted neurons survived despite cre-expression and presumed absence of a functional *fdft1 *gene. To confirm that the *fdft1 *locus was indeed recombined, we prepared genomic DNA from two exemplary regions: the cerebellum and the pontine nuclei. Southern blot on DNA from 2 months old animals showed that about 80 % of the total cerebellar DNA was recombined and thus lacked a functional squalene synthase gene (Figure 1G). Granule cells make up approximately 80% of all cells in the cerebellum [18], and they are the only cre-expressing cells in the cerebellum of Tg(mα6-cre)B1LFR. Therefore virtually all cerebellar granule cells were targeted in our mutants and lost functional squalene synthase gene. A time course using quantitative PCR analysis provided equivalent results with 57 +/- 9 % (mean +/- SEM, n = 3) recombination at postnatal day 16 and 74 +/- 4 % (mean +/- SEM, n = 4) at one month of age. Recombination in the pontine nuclei was also assayed by quantitative PCR and was found to be 9 +/- 1 % (mean +/- SEM, n = 3) at postnatal day 16 and 13 +/- 4 % (mean +/- SEM, n = 4) at one month of age. Considering the relatively crude dissection including some neighboring tissue, this confirmed that a significant fraction of the projection neurons in the pontine nuclei lost a functional squalene synthase gene.

Western blot analysis did not reveal a significant difference in the amount of squalene synthase protein between mutants and controls in extracts from total cerebellum or the pontine nuclei, neither at P16 (Figure 1H), nor at one or two months of age (data not shown). Thus, either the affected neurons only contribute little to the total pool of squalene synthase protein and thus cholesterol synthesizing capacity, or other cells might have upregulated the *fdft1 *gene to compensate.

**Figure 2:**
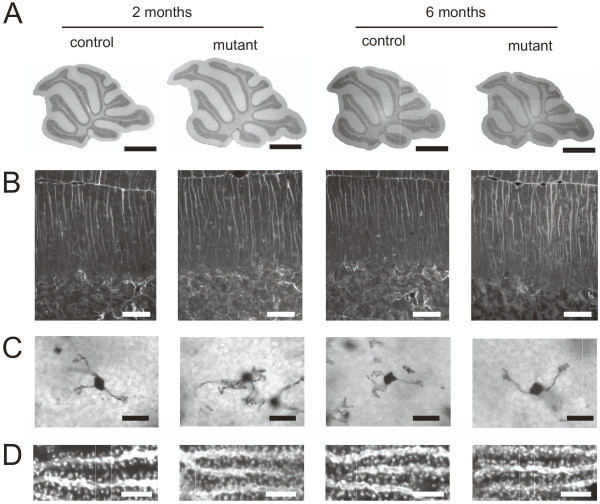
**Cerebella from mutant and control animals look indistinguishable at the age of two and six months**. (A) Normal lobulation pattern. Sagittal paraffin sections stained with hematoxylin/eosin, scale bar = 1 mm. (B) Bergmann glia are normal and no astrogliosis is evident. GFAP stained paraffin sections (lobule 8); scale bar = 50 μm. (C) Granule cell dendrites are normally developed. Golgi impregnated sections showing individual granule cells; scale bar = 20 μm. (D) Purkinje cell spines are normal; single confocal planes of Purkinje cell dendrites stained for calbindin, scale bar = 5 μm.

To assess whether lack of neuronal cholesterol synthesis affects the morphology of the targeted brain area, we performed histological analysis of cerebellum and the brain stem from mutants and controls at the age of two months (when cerebellar development is complete) and six months (to study signs of long term neurodegeneration). The size and lobulation pattern of the cerebella was unaffected (Figure 2A). Staining for glial fibrillary acidic protein (GFAP) revealed a normal glial scaffold (Figure 2B), indicating that the Bergmann glia were not disturbed. Furthermore, the GFAP staining showed no sign of astrogliosis in the cerebellum, suggesting no drastic tissue damage. Staining the tissue for Mac-3, a marker of activated microglia, or analysis of hematoxylin/eosin stained sections also revealed no sign of inflammation or cell death (data not shown). Golgi impregnation was used to reveal the shape of individual granule cells (Figure 2C). Normal dendrites with well-developed claw-like ends were present in mutants and controls. Staining sections with the granule cell maturation marker GABA_A_-α6 revealed no difference between mutants and controls, indicating a normal maturation of the mutant granule cells (data not shown). Purkinje cell spines, the postsynaptic target of cerebellar granule cell axons, were visualized by immunolabeling of the Ca^2+^-binding protein calbindin. Purkinje cell dendrites and spiny branchlets were completely normal (Figure 2D). Purkinje cells are unique in that they form numerous spines even in the absence of parallel fiber innervation [19]. To ensure that the spines were properly innervated by parallel fibers, we analyzed control and mutant mice by electron microscopy. Ultrastructural analysis of granule cell to Purkinje cell synapses did not reveal any obvious differences between mutants and controls (Figure 4A). Normal synaptic clefts and synaptic vesicles were observed. There were no "multiple synaptic units" (a single parallel fiber varicosity contacting multiple spines), as it has been described in other cerebellar mutants with cerebellar granule cell defects [19,20]. From these results, we conclude that granule cells develop normally and do not show any signs of degeneration even at the age of six months.

**Figure 3:**
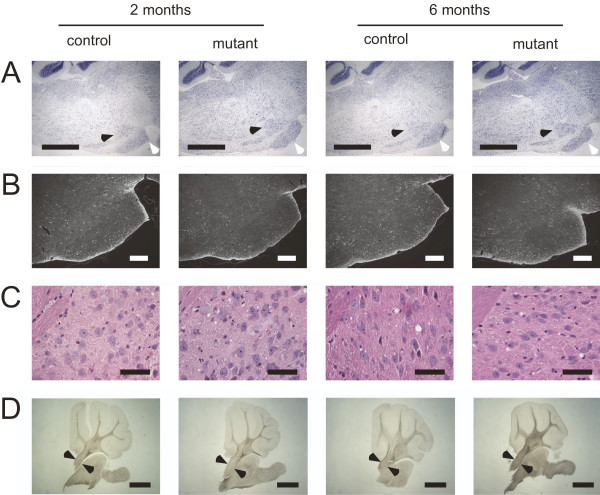
**The pontine nuclei from mutant and control animals look indistinguishable at the age of two and six months**. (A) The pontine nuclei (white arrow head) and the reticulotegmental nucleus of the pons (black arrow head) appear normal (Nissl stained paraffin sections), scale bar = 1 mm. (B) No astrogliosis is evident on paraffin sections stained for GFAP, scale bar = 200 μm. (C) The neurons in the pontine nuclei appear normal by hematoxylin eosin stain, scale bar = 50 μm. (D) The middle cerebellar peduncle (between arrow heads), the tract carrying the axons from the pontine nuclei to the cerebellar hemispheres, appear normal in size (unstained 50 μm sagittal sections), scale bar = 1 mm.

Analysis of the brain stem revealed a normal size and location of the pontine nuclei and the reticulotegmental nucleus of the pons (Figure 3A). Staining for GFAP revealed no signs of astrogliosis (Figure 3B). Examination of hematoxylin and eosin stained sections revealed normal cytoarchitecture and fiber tract distribution in the pontine nuclei (Figure 3C and data not shown). The axons from the pontine nuclei and the reticulotegmental nucleus of the pons project to the cerebellar hemispheres as a thick bundle, the middle cerebellar peduncle. The size of the middle cerebellar peduncle was unchanged (Figure 3D), indicating that the axons were projecting normally to their target.

**Figure 4:**
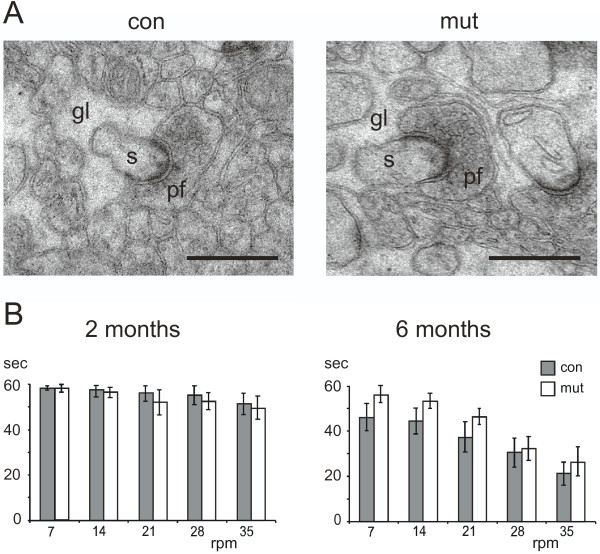
**Synapses appear normal**. (A) The ultrastructure of parallel fiber to Purkinje cell synapses was normal by electron microscopy between 2 months old control (con) and mutant (mut) animals. Parallel fibers (pf) synapse on Purkinje cell spines (s) that are fully enwrapped in Bergmann glial processes (gl). Scale bar = 500 nm. (B) Mutants performed normally on a Rotarod. The test was performed as described [27] at fixed speed levels (rpm: revolutions per minute) at the age of two and six months. The latency to fall was measured in seconds (sec) and the experiment was terminated after 60 s. The mean +/- (standard error) is shown (n>10 animals per group).

Since the cerebellum is required for motor coordination, cerebellar defects lead to characteristic deficits in motor coordination tasks like the Rotarod [21]. Here, mutant and control animals performed equally well at all speed levels tested, indicating proper cerebellar function (Figure 4B).

## Discussion

Using mouse genetics, we have generated a novel mouse mutant lacking cellular cholesterol synthesis in defined populations of adult neurons, the cerebellar granule cells and neurons in some precerebellar nuclei. These mice were phenotypically indistinguishable from controls and showed no motor deficits. This is particularly remarkable since two "links" of the cerebellar circuit were affected: a subset of mossy fibers (including fibers from the pontine nuclei and from the reticulotegmental nucleus of the pons) as well as all granule cells. Histological and ultrastructural analysis of adult mice failed to reveal any signs of neurodegeneration or inflammation. Thus, some adult neurons do not require cell autonomous cholesterol synthesis.

Genetic or pharmacological perturbation of cholesterol metabolism is known to exert devastating effects on developing neurons both in vivo and in vitro. Depending on the time point, this includes neural tube closure defects, holoprosencephaly, neuronal cell death, reduction of dendritic outgrowth, and the inhibition of dendritic and synaptic maturation [1,13,22]. Only by choosing a cre line that recombines fdft1^flox/flox ^after the targeted neurons have reached their final position, and after axons and dendrites have been formed, it was possible to exclude such developmental defects. Synaptogenesis and synaptic maturation, however, continues for several weeks after onset of cre-recombination [18]. Although we cannot exclude subtle defects of these mutants in synaptic function, which can only be detected using electrophysiological recordings or refined motor learning tasks [23], we have documented a remarkably intact development and maintenance of cerebellar granule cells and neurons in the pontine nuclei in vivo with absence of any degeneration or cell death in the adult cerebellum or brain stem. Since other defects in cholesterol metabolism lead to neurodegeneration (i.e. Niemann-Pick Type C disease, Alzheimer's disease [7]), the critical difference of our transgenic model is that only a defined subgroup of neurons is affected, whereas other neurons, oligodendrocytes and astrocytes cells maintained cholesterol synthesis.

Both neuronal subtypes analyzed in this study are glutamatergic neurons derived form the rhombic lip, but they represent two very different size classes of neurons: cerebellar granule cells are among the smallest neurons in the brain and have simple dendrites and unmyelinated axons, whereas the neurons in the pontine nuclei are large, have complex dendrites, and long myelinated axons. Since these two very different populations of neurons show a similar tolerance for loss of the *fdft1 *gene, this might be a general feature of adult neurons. However, the adult cerebellum has only about half the steady state level of cholesterol production compared to the forebrain, indicating that the cholesterol requirements might differ between different brain regions [24]. A further indication for diversity comes from culture experiments where hippocampal neurons have different cholesterol requirements compared to cortical neurons [25].

Our results support the hypothesis that adult neurons reduce cholesterol production, and that glial cells take over this task. Our western blot results even indicate that adult granule cells barely contribute to the cerebellar pool of squalene synthase, i.e. cholesterol synthesis. Alternatively, other cell types might have upregulated the steady state level of squalene synthase protein to compensate. In the pontine nuclei, the small fraction of recombined cells (only about 10 %) in the dissected tissue might make a reduction of squalene synthase protein difficult to detect. When squalene synthase was eliminated in myelinating glia, the cholesterol transport protein ApoE and its receptor LRP were upregulated in the spinal cord, suggesting that cholesterol uptake by oligodendrocytes was increased to compensate for the loss of cholesterol synthesizing capacity [11]. We could not detect an upregulation of LRP in the cerebellum (data not shown). This might be a further indication that adult neurons already express sufficient amounts of LRP to import cholesterol from ApoE-containing lipoprotein particles, or they might utilize a different cholesterol uptake system. Oligodendrocytes and astrocytes are the most likely candidates responsible for providing cholesterol in a paracrine fashion. Astrocytes have been shown to secrete cholesterol in the form of ApoE-containing particles [12]. Indeed, cerebrospinal fluid contains about 8 μg/ml cholesterol [26], a concentration sufficient to sustain full neuronal growth and maturation *in vitro *[13].

## Conclusion

Our finding that adult neurons can survive and function in the absence of cholesterol synthesis demonstrates the importance of horizontal cholesterol flux in vivo. It also highlights the importance of glial cells on brain cholesterol homeostasis.

## Methods

### Mice and behavior

All animal experiments were carried out in compliance with animal policies of the State of Niedersachsen, Germany. Animals were maintained on a mixed genetic background (C57/Bl6, Sv129). Motor behavior analysis was performed on an accelerating Rotarod (Hugo Basile 7650) at fixed speed levels as described [27].

### Southern Blot

20 μg of total cerebellar DNA were digested with HindIII, and a radiolabeled Southernblot was performed using standard procedures. The template was a 343 bp fragment of intron IV of the *fdft1 *gene, generated by PCR from genomic DNA using the primers GAGATGGACGTCCTCCCAGCCTGAGGC and GCAGCCTACCAACTCCTAGCCTGGC. The fragment of the flox allele migrates at 2.9 kb, the recombined allele at 6.1 kb.

### Quantitative PCR

Quantitative PCR was performed using 10 ng of target DNA in a 12.5 μl assay using SYBR Green PCR Master Mix (Applied Biosystems 4367659) on an Applied Biosystems 7500 Fast real time PCR system in duplicates. The standard curve was generated using DNA from heterozygous knockout animals fdft1^flox/rec ^(50 % recombination) and fdft1^flox/flox ^animals (0 % recombination). To achieve a standard curve between 50 % and 100 % recombination, 20 ng of fdft1^flox/rec ^DNA were set as 100 % recombination, and all samples falling in that range were supplemented with 10 ng of "dummy DNA" (fdft1^flox/flox^) to equalize the amount of DNA. The values were standardized to an independent genomic marker to correct for small differences in the amount of DNA. Primers AAGGCTGGATCCGTCGAGGTG and GTCTTCAAGAATTCCGATCATATTCA amplify a 112 bp band specific for the recombined allele.

### Western Blot

10 μg of extract were separated by SDS PAGE and blotted onto nitrocellulose. The blot was probed with mouse anti squalene synthase (BD Transduction Laboratories 611808, 1:1000), and later reprobed with mouse anti glyceraldeyde-3-phosphate dehydrogenase (GAPDH; Advanced Immunochemicals #RGM2, 1:5000). Bands were detected using a fluorescent secondary antibody and scanned on an Odyssey infrared scanner (LI-COR Biosciences).

### Histology and immunohistochemistry

For all histological analyses, three mutants and three control littermates were analyzed. Mice were anesthetized with Halothane and perfused intracardially with 15 ml of Hanks' balanced salt solution (HBSS) (PAA Laboratories H15-010), followed by 50 ml of 4 % formaldehyde in 100 mM phosphate buffer (PB). Brains were postfixed in 4 % formaldehyde (in PB) overnight and then either embedded in paraffin or stored in PBS. For sectioning on the vibratome (Leica VT 1000 S), sagittal halves of brains were embedded in 2 % low-melt agarose and cut at 50 μm. Vibratome sections were stained free floating. Paraffin embedded tissue was sectioned at 5 μm on a sliding microtome (Microm HM400). Sections were stained with hematoxylin/eosin, for Nissl or immunohistochemically and photographed on a Zeiss Axiophot or a Leica DMRXA. The cerebellar overview bright field pictures were manually cleared of background and brightness/contrast on all pictures was optimized using the software Deneba Canvas. Confocal pictures were taken on a Zeiss LSM 510 Meta microscope. Antibodies used: rabbit anti S100β (SWANT code 37, 1:1000), mouse anti GFP (Molecular Probes A11120, 1:500), mouse anti GFAP (Novocastra NCL-GFAP-GA5, 1:200), rabbit anti GABA_A_α6 (Chemicon AB5610, 1:500), rat anti Mac-3 (Pharmingen 01781, 1:5000), rabbit anti calbindin (Swant CB-38a 1:5000). Golgi impregnation was performed using a Rapid Golgi Stain Kit (FD Neuro Technologies PK401) on entire halves of freshly dissected cerebellum.

### Electron microscopy

Animals were anesthetized with Halothane and perfused intracardially with 15 ml of HBSS, followed by 50 ml of fixative (2.5 % glutaraldehyde, 4 % paraformaldehyde in phosphate buffered saline [28]), and the whole cerebellum was postfixed overnight in fixative. Midsagittal 1 mm slices of whole cerebellum were embedded in Epon, blocks were trimmed to contain lobules 6/7. Ultrathin sections were contrasted with 1 % uranyl acetate and lead citrate [29]. Parallel fiber to Purkinje cell synapses were identified by their morphology [30]. Pictures were taken on a LEO 912AB electron microscope (Zeiss, Oberkochen, Germany) with an on-axis 2048 × 2048 CCD-camera (Proscan, Scheuring, Germany) at 10 000x, and digitally enhanced using the program ImageJ [31].

## Authors' contributions

UF performed animal breeding, behavior analysis, histololgy, biochemistry experiments, and drafted the manuscript. GS generated and characterized the fdft1^flox ^mouse, provided important scientific input, and revised the manuscript. LX performed cell culture experiments, which provided conceptual input. WM performed the electronmicroscopic analysis. KAN provided essential scientific input and revised the manuscript. All authors read and approved the final manuscript.
